# Supplementary material for: Psychotherapeutic interventions for burns patients and the potential use with Stevens-Johnson syndrome and toxic epidermal necrolysis patients: A systematic integrative review
Source: PLoS One. 2022 Jun 27;17(6):e0270424. doi: 10.1371/journal.pone.0270424 (PMC9236256; doi:10.1371/journal.pone.0270424)
Supplement: S1 Table — (DOCX) [file pone.0270424.s002.docx]

**SI Table: Medline search strategy**

|  | **MEDLINE SEARCH** |
| --- | --- |
| **#** | **Query** |
| S1 | (MH "Stevens-Johnson Syndrome") |
| S2 | TI "stevens johnson syndrome" OR "stevens-johnson syndrome" OR SJS OR TI "toxic epidermal necrolysis" OR TI "toxic epidermal necrolyses" OR TI ( lyell W1 (syndrome* or disease*) ) |
| S3 | AB "stevens johnson syndrome" OR "stevens-johnson syndrome" OR SJS OR AB "toxic epidermal necrolysis" OR AB "toxic epidermal necrolyses" OR AB ( lyell W1 (syndrome* or disease*) ) |
| S4 | TX ( psych* N3 (care or manag* or intervention* or strateg* or technique* or approach or approaches) ) OR TX ( psych* N3 (support or nursing or nurse*) ) OR TX ( psych* N3 (accompaniment or accompagnement or treatment or treating) ) OR TX ( psychotherap* N3 (care or manag* or intervention* or strateg* or technique* or approach or approaches) ) OR TX ( psychotherap* N3 (support or nursing or nurse*) ) OR TX ( psychotherap* N3 (accompaniment or accompagnement or treatment or treating) ) |
| S5 | TX ( counselling or counsellor ) OR TX ( "patient centered nursing" OR "patient centred nursing" ) OR TX ( "patient centered care" OR "patient centred care" ) |
| S6 | TX ( "affirmative therapy" or "affirmative therapies" OR "cognitive therapy" or "cognitive therapies" OR "emotion focused therapy" or "emotion focused therapies" ) OR TX "emotional support" OR TX diary N1 writaring OR TX distract* N1 technique* OR TX ( meditation or mindfulness or hypnosis ) |
| S7 | TX ( anxiety N2 (manag* or intervention* or support or technique*) ) OR TX ( ptsd N2 (manag* or intervention* or support or technique*) ) OR TX ( "post traumatic stress disorder" N2 (manag* or intervention* or support or technique*) ) |
| S8 | TX ( "self esteem" N2 (manag* or intervention* or support or technique*) ) OR TX ( "self-esteem" N2 (manag* or intervention* or support or technique*) ) OR TX ( "body image" N2 (manag* or intervention* or support or technique*) ) OR TX ( panic N2 (manag* or intervention* or support or technique*) ) OR TX ( stress N2 (manag* or intervention* or support or technique*) ) OR TX ( fear N2 (manag* or intervention* or support or technique*) ) |
| S9 | TX ( premorbid N2 (manag* or intervention* or support or technique*) ) OR TX ( "pre-morbid" N2 (manag* or intervention* or support or technique*) ) OR TX ( "pre morbid" N2 (manag* or intervention* or support or technique*) ) |
| S10 | S1 OR S2 OR S3 |
| S11 | S4 OR S5 OR S6 OR S7 OR S8 OR S9 |
| S12 | S10 AND S11 |
| **S13** | **S10 AND S11** |
| S14 | (MM "Burns") OR (MM "Burns, Chemical") OR (MH "Burns, Electric") |
| S15 | ( AB burn? N2 patient? OR AB sever* N1 burn? ) OR ( TI burn? N2 patient? OR TI sever* N1 burn? ) |
| S16 | (MH "Intensive Care Units") OR (MH "Burn Units") OR (MH "Critical Care") OR (MH "Critical Care Nursing") |
| S17 | ( TI ( "intensive care unit" OR ICU OR "intensive therapy unit" OR ITU OR "critical care" OR "High dependency" OR "post anaesthesia care unit" OR "post anesthesia care unit" OR PACU OR "recovery room" ) ) OR ( AB ( "intensive care unit" OR ICU OR "intensive therapy unit" OR ITU OR "critical care" OR "High dependency" OR "post anaesthesia care unit" OR "post anesthesia care unit" OR PACU OR "recovery room" ) ) |
| S18 | S14 OR S15 |
| S19 | TI ( burn? W1 (center? or centre? or unit?) ) OR AB ( burn? W1 (center? or centre? or unit?) ) |
| S20 | TI ( "acute phase" or "short term" or "short-term" ) OR AB ( "acute phase" or "short term" or "short-term" ) |
| S21 | S16 OR S17 OR S19 OR S20 |
| **S22** | **S11 AND S18 AND S21** |
