# Supplementary material for: Psychotherapeutic interventions for burns patients and the potential use with Stevens-Johnson syndrome and toxic epidermal necrolysis patients: A systematic integrative review
Source: PLoS One. 2022 Jun 27;17(6):e0270424. doi: 10.1371/journal.pone.0270424 (PMC9236256; doi:10.1371/journal.pone.0270424)
Supplement: Attachment — Submitted filename: Rebuttal Letter.pdf [file pone.0270424.s005.pdf]

Reviewers

PLOSONE

April 21st, 2022

Re: Paper Re-Submission

PONE-D-21-37340

Psychotherapeutic interventions for burns patients and the potential use with Stevens-Johnson syndrome and toxic epidermal necrolysis patients: a systematic integrative review

Dear Reviewers,

I wish to thank you both for taking the time to review our systematic review.

We found the feedback and comments to be most helpful and insightful. In light of these, we have revised the manuscript. Please see below, a supporting document outlining the details of the revisions.

Once again, many thanks for your comments and advice. I look forward to hearing from you.

Yours sincerely

Dr Pauline O'Reilly
Senior Lecturer
Department of Nursing and Midwifery,
University of Limerick,
Limerick,
Ireland
Email: Pauline.OReilly@ul.ie
On behalf of the research team

Table of Revision

Reference number: PONE-D-21-37340 Psychotherapeutic interventions for burns patients and the potential use with Stevens-Johnson syndrome and toxic epidermal necrolysis patients: a systematic integrative review. PLOS ONE

Editor

Please ensure that your manuscript meets PLOS ONE's style requirements, including those for file naming.

Response

The article was checked to ensure it met PLOSONE requirements. Headings were revised and figures were uploaded unto PACE and now meet the journal requirements. All references were reviewed and amended where necessary, to meet PLOSONE referencing criteria**.**

Editor

Changes to Data Availability statement

We note that you have stated that you will provide repository information for your data at acceptance. Should your manuscript be accepted for publication, we will hold it until you provide the relevant accession numbers or DOIs necessary to access your data. If you wish to make changes to your Data Availability statement, please describe these changes in your cover letter and we will update your Data Availability statement to reflect the information you provide.

Response

In relation to the Data Availability statement, please amend to -all material relating to this systematic review are available within the article and supporting information files.

Reviewer: #1

Thank you for allowing me to review the manuscript entitled: Psychotherapeutic interventions for burns patients and the potential use with Stevens-Johnson syndrome and toxic epidermal necrolysis patients: a systematic integrative review. This is an interesting review and how it was conducted. The question that comes to mind is does it

add anything new to the body of knowledge as SJS/TEN patients are often cared for a burn intensive care unit as SJS/TEN mirrors the requirements of burn patients due to insensible losses through wounds, however fluid requirements are reduced in burn patients with similar TBSA involvement.

Response

Included referenced evidence on this point, see Page 5&6, Line 192-209

Reviewer: #1

The authors use both PTS and PTSD. For consistency use one acronym.

Response

PTS was used when a diagnostic instrument wasn't used (the patients had symptoms of post-traumatic stress, but they were not sufficient to meet the full criteria for a disorder of PTSD). PTSD was used when a diagnosis had been given using a recognised instrument (the symptoms are of sufficient intensity and frequency to warrant a diagnosis - meeting the accepted criteria, whether that is according to DSM or ICD criteria, DSM or ICD - depending in the country/continent where the research was conducted).

Reviewer: #1

The referencing formatting intext needs review as shown here: [39, 45, 47] [40, 41, 46, 48, 50] [42, 49].

Response

All references were reviewed and amended where necessary, to meet PLOSONE referencing criteria.

Reviewer: #1

There is a missing arrow on the SJS/TEN PRISMA.

Response

Amended – arrow included

Reviewer: #1

Tables are difficult to read due to portrait presentation. Tables need to be formatted accordingly in Landscape. The author, year and country should be combined in one column. The first column with the numbers should be deleted. Strongly suggest the authors review how other review tables are resented.

Response

The tables were revised and are presented in Landscape format. In relation to Tables 2 & 3 – the first column was deleted, and the author, year and country were combined into one column. Please see amended tables, Pages 12-17.

Reviewer: #1

Some key literature has been missed and should be included for the currency of the review:

Schneider, J. A., & Cohen, P. R. (2017). Stevens-Johnson Syndrome and Toxic Epidermal Necrolysis: A Concise Review with a Comprehensive Summary of Therapeutic Interventions Emphasizing Supportive Measures. Advances in therapy, 34(6), 1235–1244. https://doi.org/10.1007/s12325-017-0530-y

Shanbhag, S. S., Chodosh, J., Fathy, C., Goverman, J., Mitchell, C., & Saeed, H. N. (2020). Multidisciplinary care in Stevens-Johnson syndrome. Therapeutic Advances in Chronic Disease. https://doi.org/10.1177/2040622319894469

Frantz, R., Huang, S., Are, A., & Motaparthi, K. (2021). Stevens-Johnson Syndrome and Toxic Epidermal Necrolysis: A Review of Diagnosis and Management. Medicina (Kaunas, Lithuania), 57(9), 895. https://doi.org/10.3390/medicina57090895

Response

Amended. This key literature has been included in the Introduction and the Discussion. Please see: Page 5; Line 173-175.

Page 5&6; Line 192-209.

Page 30&31; Line 943-963

Reviewer: #2

The aim of this systematic integrative review was to synthesize the evidence relating to

Abstract Aims and objectives:

Add in the word ‘adult’

psychotherapeutic interventions used with adult burns patients and patients with SJS/TENs.

Response

Amended. The word ‘adult’ was included in the aim. Please see: Page 2, Line 49-51

Reviewer: #2

Abstract -Results

Remove the word ‘dearth’ and clearly state that there was no evidence for psych interventions in SJS/TENs.

Response

Amended. The word ‘Dearth’ was replaced with ‘no evidence’. Please see: Page 3, Line 72.

Reviewer: #2

Mostly throughout the manuscript the intext reference falls after the full stop at the end of a sentence or after a comma mid-sentence instead of before the punctuation, e.g., line 90, 98, 105, 115. Please correct.

Response

All references were reviewed and amended where necessary, to meet PLOSONE referencing criteria. All intext references are now placed before punctuation.

Reviewer: #2

Methods: The review question should state burn patient ‘either/or’ SJS-TENs patients not ‘both/and’:

‘What is the evidence on the psychotherapeutic interventions which have been used with (either) both adult burns patients or and patients with SJS/TEN, during the acute stage of the illness, to reduce symptoms aligned to PTS and improve quality of life?’

Response

This review question was revised. Please see: Page 8, Line 248.

Reviewer: #2

State how you completed the QA process as a group and list author initials.

Response

This section was amended to include the process of assessment and the initials of the authors who appraised the studies.

Please see: Page 10, Line 300-305.

Reviewer: #2

Table 2: A lot of the information can be reduced without losing context. I would probably present the qualitative studies in separate table, purely because the information presented is wordier and therefore from a formatting point of view will look better separate. Perhaps reduce the font/line separation etc. similar to table 3 to allow the columns to be read more easily, although this might be an editing task by the journal.

• Do you need paper number in column 1?

• Add the country under the authors names and add the reference

• Summarise the aim

• Add in outcome measures used - this column appears to be blank for all papers. Would be interesting to add the time post-burn at which the outcomes are measured.

• Summarise key findings (with stats if available).

• Reorder columns: Author/ country; aim; study design; study participants; outcome measures; analyses; key findings.

Response

The table was amended as per suggestions. There are now two tables viz. Table 2 presents the quantitative papers and Table 3 presents the qualitative papers.

Column 1 was removed.

The country, authors' names and reference are now in one column.

All aims were summarized.

Outcome measures and references were added. However, it was difficult to determine the time post burn at which the outcomes were measured for most of the studies.

Key findings were summarized, and statistics were included were available.

Columns were reordered as per recommendation.

Please see Table 2&3:

Pages 12-17

Reviewer: #2

Line 253: Please change: 'Burn Specific Health Scale – Brief' not 'Burning Specific Health Scale'

Response

Spelling error amended from Burning to Burn. Please see: Page 19, Line 515.

Reviewer: #2

You are interested in long term psychological sequelae after SJS/TENs but not many of these studies assesses the long-term effects of the interventions found, with some exceptions e.g., patients who receive the multimedia training are assessed at 3- and 6-months post intervention. In fact, theme 2 treatments seem to address the long-term recovery more than the theme 1 treatments. This would be good to explore in the discussion, particularly with regards to the need for longitudinal research in the future.

Response

A section was included outlining the need for more longitudinal studies. Please see: Page 30&31, Line 962-982
