# Supplementary material for: Psychotherapeutic interventions for burns patients and the potential use with Stevens-Johnson syndrome and toxic epidermal necrolysis patients: A systematic integrative review
Source: PLoS One. 2022 Jun 27;17(6):e0270424. doi: 10.1371/journal.pone.0270424 (PMC9236256; doi:10.1371/journal.pone.0270424)
Supplement: Attachment — Submitted filename: Response To Reviewers.pdf [file pone.0270424.s006.pdf]

UNIVERSITY OF LIMERICK
OLLSCOIL LUIMNIGH

Table of Revision
Reference number: PONE-D-21-37340R1 Psychotherapeutic interventions for burns patients and the potential use with Stevens-Johnson syndrome and toxic epidermal necrolysis patients: a systematic integrative review. PLOS ONE

Journal Requirements

Please review your reference list to ensure that it is complete and correct. If you have cited papers that have been retracted, please include the rationale for doing so in the manuscript text or remove these references and replace them with relevant current references. Any changes to the reference list should be mentioned in the rebuttal letter that accompanies your revised manuscript. If you need to cite a retracted article, indicate the article's retracted status in the References list and also include a citation and full reference for the retraction notice.

Response

The article was checked to ensure it met PLOSONE requirements. In the previous revised article, all references were reviewed and amended where necessary, to meet PLOSONE referencing criteria. No reference was retracted. With the tracked changes, some references are under the heading 'deleted', however, the corrected versions of the references were updated and included. All references were rechecked.

Reviewer: #1

PTSD and PTS are not presented or defined as per formatting requirements as all acronyms need to be formatted accordingly – Post Traumatic Stress Disorder (PTSD) when used for the first time then PTSD thereafter.

Response

Amended: Post-Traumatic Stress Disorder was written in full initially and the acronym PTSD was used subsequently.

Please see Pg 5; Line 111

Post Traumatic Symptoms had already been written in full and the acronym PTS used thereafter.

Please see Pg 4; Line 97-98

Reviewer: #1

Line 134 there are 2 full stops in at the end of the sentence.

Response

Amended: Extra full stop removed.

Please see Pg 5; Line 125

Reviewer: #1

There is inconsistency with how et al is presented when the author is used first et al., & et al. is used throughout.

Response

Amended: The inconsistency with et al., was revised and amended. There were 18 revisions. Please see Pg 4; Line 94; Pg 4; Line 101; Pg 19; Line 265; Pg 19; Line 271; Pg 20; Line 279; Pg 21; Line 307; Pg 22; Line 341 – 348; Pg 24; Line 373 – 376; Pg 25; Line 406 & 410; Pg 28; Line 499 and Pg 32; Line 577.

Reviewer: #1

viz is throughout the manuscript no idea what it is referring to as could not find an explanation.

Response

Amended – the use of 'vis.' was revised throughout the article and removed were appropriate.

Please see Pg 18; Line 246; Pg 20; Line 279; Pg 26; Line 433; Pg 28; Lines 470, 483, 487, Pg 29; Line 498 & 508; Pg 30; Line 530 and Pg 31; Line 553.

Reviewer: #1

When using direct quotes from the included qualitative studies, I believe a page number may be required?

Response

Amended: The page number was used with each quotation.

Please see Pg 27; Line 457; Pg 27; Line 461 and Pg 27; Line 468.

Thank you.
