# Supplementary material for: Psychotherapeutic interventions for burns patients and the potential use with Stevens-Johnson syndrome and toxic epidermal necrolysis patients: A systematic integrative review
Source: PLoS One. 2022 Jun 27;17(6):e0270424. doi: 10.1371/journal.pone.0270424 (PMC9236256; doi:10.1371/journal.pone.0270424)
Supplement: Attachment — Submitted filename: Psychotherapeutic interventions for burn patients and the potential use with SJSTENs patients.docx [file pone.0270424.s004.docx]

Review – Psychotherapeutic interventions for burn patients and the potential use with SJS/TENs patients: a systematic integrative review.

To the best of my understanding the criteria have been met:

1. The study presents the results of original research.
   2. Results reported have not been published elsewhere.
   3. Experiments, statistics, and other analyses are performed to a high technical standard and are described in sufficient detail.
   4. Conclusions are presented in an appropriate fashion and are supported by the data.
   5. The article is presented in an intelligible fashion and is written in standard English.
   6. The research meets all applicable standards for the ethics of experimentation and research integrity.
   7. The article adheres to appropriate reporting guidelines and community standards for data availability.

This systematic integrative review assesses the published evidence of psychotherapeutic interventions to alleviate psychological distress following burn injury, in the absence of published evidence of psychotherapeutic interventions for Stevens-Johnson syndrome and TEN, on the basis that is it reasonable to suggest that the psychological sequelae of the condition are similar.

This is a well written and clearly presented manuscript, which I believe meets the journal criteria and can be accepted for publication with some minor changes. I like the fact that you were able to include papers from the French language as well as English.

Abstract Aims and objectives

Add in the word ‘adult’

The aim of this systematic integrative review was to synthesize the evidence relating to psychotherapeutic interventions used with ***adult*** burns patients and patients with SJS/TENs.

Abstract Results

Remove the word ‘dearth’ and clearly state that there was **no** evidence for psych interventions in SJS/TENs.

Introduction

Mostly throughout the manuscript the intext reference falls after the full stop at the end of a sentence or after a comma mid-sentence instead of before the punctuation; eg line 90, 98, 105, 115. Please correct.

Methods

The review question should state burn patient ‘either/or’ SJS-TENs patients not ‘both/and’:

‘What is the evidence on the psychotherapeutic 150 interventions which have been used with (either)  ~~both~~ adult burns patients **or** ~~and~~ patients with 151 SJS/TEN, during the acute stage of the illness, to reduce symptoms aligned to PTS and 152 improve quality of life?’.

State how you completed the QA process as a group and list author initials.

Table 2: A lot of the information can be reduced without losing context. I would probably present the qualitative studies in separate table, purely because the information presented is wordier and therefore from a formatting point of view will look better separate. Perhaps reduce the font/line separation etc similar to table 3 to allow the columns to be read more easily, although this might be an editing task by the journal.

- Do you need paper number in column 1?
- Add the country under the authors names and add the reference
- Summarise the aim
- Add in outcome measures used - this column appears to be blank for all papers. Would bbe interesting to add the time post-burn at which the outcomes are measured.
- Summarise key findings (with stats if available).
- Reorder columns: Author/ country; aim; study design; study participants; outcome measures; analyses; key findings.

Line 253: Please change: ‘Burn Specific Health Scale – Brief’ not ‘Burning Specific Health Scale’

Discussion:

You are interested in long term psychological sequelae after SJS/TENs but not many of these studies assesses the long term effects of the interventions found, with some exceptions eg patients who receive the multimedia training are assessed at 3 and 6 months post intervention. In fact, theme 2 treatments seem to address the long term recovery more than the theme 1 treatments. This would be good to explore in the discussion, particularly with regards to the need for longitudinal research in the future.

I have no competing interests to declare.

Well done, this is looking good. Hopefully my suggestions will take it up a notch, too.
